# The Repeat Sequences and Elevated Substitution Rates of the Chloroplast *accD* Gene in Cupressophytes

**DOI:** 10.3389/fpls.2018.00533

**Published:** 2018-04-20

**Authors:** Jia Li, Yingjuan Su, Ting Wang

**Affiliations:** ^1^Department of Life Sciences, Shaanxi Xueqian Normal University, Xi’an, China; ^2^School of Life Sciences, Sun Yat-sen University, Guangzhou, China; ^3^Research Institute of Sun Yat-sen University, Shenzhen, China; ^4^College of Life Science, South China Agricultural University, Guangzhou, China

**Keywords:** *accD*, repeat sequences, substitution rates, rearrangement, cupressophytes

## Abstract

The plastid *accD* gene encodes a subunit of the acetyl-CoA carboxylase (ACCase) enzyme. The length of *accD* gene has been supposed to expand in *Cryptomeria japonica*, *Taiwania cryptomerioides*, *Cephalotaxus*, *Taxus chinensis*, and *Podocarpus lambertii*, and the main reason for this phenomenon was the existence of tandemly repeated sequences. However, it is still unknown whether the *accD* gene length in other cupressophytes has expanded. Here, in order to investigate how widespread this phenomenon was, 18 *accD* sequences and its surrounding regions of cupressophyte were sequenced and analyzed. Together with 39 GenBank sequence data, our taxon sampling covered all the extant gymnosperm orders. The repetitive elements and substitution rates of *accD* among 57 gymnosperm species were analyzed, the results show: (1) Reading frame length of *accD* gene in 18 cupressophytes species has also expanded. (2) Many repetitive elements were identified in *accD* gene of cupressophyte lineages. (3) The synonymous and non-synonymous substitution rates of *accD* were accelerated in cupressophytes. (4) *accD* was located in rearrangement endpoints. These results suggested that repetitive elements may mediate the chloroplast genome rearrangement and accelerated the substitution rates.

## Introduction

Cupressophytes, also called non-Pinaceae conifers, comprise about 380 species in 58 genera of five families: Araucariaceae, Podocarpaceae, Sciadopityaceae, Taxaceae (including Cephalotaxaceae), and Cupressaceae (including Taxodiaceae) (Christenhusz et al., 2011). Most species of Araucariaceae and Podocarpaceae are usually distributed in Southern Hemisphere, while other three families are located in the Northern Hemisphere. Some of the cupressophytes species are of economic and ecological value to humans. For instance, most species of Cupressaceae are valued for the production of timbers or ornamentals. The secondary metabolite paclitaxel (taxol) extracted from the bark of *Taxus* is a chemotherapy drug to treat ovarian and breast cancer.

Dispersed repetitive DNA sequences are scattered throughout the chloroplast genome. Most of the studies concentrate on detecting repeat sequences on a chloroplast genome-wide degree (Saski et al., 2005; Guo et al., 2007; Haberle et al., 2008; Tangphatsornruang et al., 2011); while there are only very few reports about the presence and structure of the repetitive DNA of a specific gene among many lineages (Hipkins et al., 1995; Erixon and Oxelman, 2008). Some repeats exist in the coding regions of chloroplast gene. For instance, *ycf1* and *ycf2* in *Panax ginseng* (Zhao et al., 2014), *Taxus chinensis* (Zhang et al., 2014), *Podocarpus lambertii* (Vieira et al., 2014), *Cephalotaxus oliveri* (Yi et al., 2013), and *Globe artichoke* (Curci et al., 2015); as well as *accD* in *T. chinensis* (Zhang et al., 2014), *P. lambertii* (Vieira et al., 2014), *Medicago truncatula* (Gurdon and Maliga, 2014), *C. oliveri* (Yi et al., 2013), *Capsicum annuum* (Jo et al., 2011), *Pisum sativum* and *Lathyrus sativus* (Magee et al., 2010) all have repeat sequences. Many studies have suggested that highly rearranged chloroplast genome generally possess a great many repetitive sequences which are associated with rearrangement endpoints, and this phenomenon has been observed in some land plants: Douglas-fir (*Pseudotsuga menziesii*) (Tsai and Strauss, 1989), *Pelargonium* (Chumley et al., 2006), *Trifolium* (Cai et al., 2008), *Trachelium* (Haberle et al., 2008), Oleaceae (Lee et al., 2007), and Asteraceae (Kim et al., 2005; Timme et al., 2007). The size and number of repeats also correlate to the extent of genome rearrangement (Guisinger et al., 2011). The genome containing the most frequency of long repeats is usually the most reorganized.

Acetyl-CoA carboxylase (ACCase) facilitate the acetyl-CoA to form malonyl-CoA and is supposed to regulate *de novo* fatty acid biosynthesis (Konishi and Sasaki, 1994; Sasaki and Nagano, 2004). Most higher plants, except for Gramineae, have two forms of ACCase: a prokaryotic type made up of several subunits in the stroma of plastids and a eukaryotic form composed of an only multifunctional polypeptide located in the cytosol (Konishi et al., 1996). The prokaryotic ACCase form is organized by the α-carboxyl transferase, the biotin carboxyl carrier, the biotin carboxylase, and the β-carboxyl transferase subunit (Gornicki et al., 1997). Except for β-carboxyl transferase was encoded by the plastid *accD* gene, other three subunit are all nucleus encoded. The plastid-localized *accD* gene is essential for leaf growth and to maintain plastid compartment in tobacco (Kode et al., 2005). Elevation of *accD* expression successfully raised the entire ACCase amount in plastids, and significantly raised the fatty acid content in tobacco leaves (Madoka et al., 2002). Furthermore, expression of *accD* was considered to be essential at the stage of embryo development in Arabidopsis (Bryant et al., 2011).

*AccD* is widely distributed in plants, including the reduced chloroplast genome of parasitic and non-photosynthetic plants (Wolfe et al., 1992; de Koning and Keeling, 2006). However, *accD* has been lost several times from the chloroplast genomes of some angiosperm lineage: Acoraceae (Goremykin et al., 2005), Poaceae (Konishi and Sasaki, 1994; Harris et al., 2012), Campanulaceae (Haberle et al., 2008), Geraniaceae (Guisinger et al., 2008), and Fabaceae (Magee et al., 2010). In Poaceae, the plastid-located prokaryotic form ACCase is functionally replaced by the nuclear-encoded eukaryotic type (Konishi et al., 1996; Gornicki et al., 1997). The loss of *accD* gene from the chloroplast genomes of Campanulaceae and Fabaceae was also consistent with an additional ACCase counterpart in the nucleus (Magee et al., 2010; Rousseau et al., 2013). In *Trifolium repens* of Fabaceae, through scanning high-throughput EST sequence data, *accD* was found to fuse with a nuclear gene for plastid lipoamide dehydrogenase (LPD2) (Magee et al., 2010); in *Trachelium caeruleum* of Campanulaceae, a transit peptide is combined with an abridged *accD* gene, which includes only 331 amino acids (Rousseau et al., 2013). In contrast to the loss of this gene among the above species, the length of *accD* gene in cupressophyte species including *Cryptomeria japonica*, *Taiwania cryptomerioides*, *Cephalotaxus wilsoniana*, *C. oliveri*, *T. chinensis*, and *P. lambertii* have diversified in an increasing direction (Hirao et al., 2008; Wu et al., 2011; Yi et al., 2013; Vieira et al., 2014; Zhang et al., 2014). The extension of the *accD* gene length is mainly caused by the insertion of large number of tandem repeated sequences in this area. But the repetitive elements of the gene are different among *Cephalotaxus*, *T. cryptomerioides*, *T. chinensis*, and *P. lambertii* (Yi et al., 2013; Vieira et al., 2014; Zhang et al., 2014). Therefore, evolutionary mechanisms underlying the occurrence of repetitive elements in cupressophyte of *accD* gene remain poorly studied. Sequence data from a wider phylogenetic breadth of cupressophytes are needed to clarify the evolutionary history of *accD* gene.

In the study of four mammalian and a bird genome, it is suggested that regions surrounding tandem repeats evolve faster than other non-repeat-containing regions (Simon and Hancock, 2009). One explanation is that regions nearby repeat sequences have evolved under weaker negative selection than the remaining region they embedded in (Djian et al., 1996; Faux et al., 2007). Another explanation is that the repeat sequences give rise to more substitutions near the flanking sequences (Huntley and Clark, 2007). Recent evidence also suggests that the insertion of repeat sequence elevated substitution rate of the entire sequence (Huntley and Clark, 2007). It is also assured that repeat sequence themselves evolves faster than their flanking sequence (Huntley and Golding, 2000). With many repeat elements in *accD*, whether the substitution rates for the repeat sequences or their flanking sequences have accelerated is unknown. To elucidate the overall evolutionary history or patterns of the repeat sequences in chloroplast genome, substitution rate pattern of *accD* gene were identified in this study.

In order to have a better insight into the evolutionary trace of *accD* in cupressophytes, in this study, we have sequenced *accD* genes from 18 cupressophytes species. The aim of this study focuses on: (1) investigating whether *accD* gene length in cupressophytes tends to increase; (2) exploring if *accD* gene in other cupressophytes species have specific repetitive elements like *Cephalotaxus*, *T. cryptomerioides*, *T. chinensis*, and *P. lambertii*; (3) determining the substitution rates pattern of *accD* in cupressophytes; (4) identifying gene order states around *accD* gene and verifying the association of repetitive elements, substitution rates and genome rearrangement.

## Materials and Methods

### Plant Sampling

Fresh leaves of 18 conifer species were sampled from Wuhan Botanical Garden, Chinese Academy of Sciences (CAS), Institute of Botany, CAS, and Sun Yat-sen University, respectively (**Table 1**). The materials used for DNA extraction were saved in silica gel.

**Table 1:** List of taxa and sequences analyzed in this study.

Family	Genus	Species	Sampling site	*accD*-GenBank	*rbcL*-GenBank	*matK*-GenBank	*AccD* gene length (codons)
Cycadaceae	*Cycas*	*Cycas taitungensis*	–	NC_009618	NC_009618	NC_009618	359
		*Cycas revoluta*	–	NC_020319	NC_020319	NC_020319	359
Ginkgoaceae	*Ginkgo*	*Ginkgo biloba*	–	NC_016986	NC_016986	NC_016986	323
Pinaceae	*Cathaya*	*Cathaya argyrophylla*	–	NC_014589	NC_014589	NC_014589	320
	*Cedrus*	*Cedrus deodara*	–	NC_014575	NC_014575	NC_014575	322
	*Keteleeria*	*Keteleeria davidiana*	–	NC_011930	NC_011930	NC_011930	323
	*Pseudotsuga*	*Pseudotsuga sinensis*	–	NC_016064	NC_016064	NC_016064	322
	*Larix*	*Larix decidua*	–	NC_016058	NC_016058	NC_016058	324
	*Picea*	*Picea morrisonicola*	–	NC_016069	NC_016069	NC_016069	326
		*Picea sitchensis*	–	NC_011152	NC_011152	NC_011152	320
		*Picea abies*	–	AJ001004	AJ001004	AJ001004	319
	*Pinus*	*Pinus thunbergii*	–	NC_001631	NC_001631	NC_001631	321
	*Strobus*	*Pinus Koraiensis*	–	NC_004677	NC_004677	NC_004677	320
		*Pinus Lambertiana*	–	NC_011156	NC_011156	NC_011156	320
		*Pinus contorta*	–	NC_011153	NC_011153	AB080921	321
		*Pinus gerardiana*	–	NC_011154	NC_011154	NC_011154	320
		*Pinus krempfii*	–	NC_011155	NC_011155	NC_011155	320
		*Pinus nelsonii*	–	NC_011159	NC_011159	NC_011159	320
		*Pinus massoniana*	–	NC_021439	NC_021439	NC_021439	321
		*Pinus taeda*	–	NC_021440	NC_021440	NC_021440	321
Podocarpaceae	*Podocarpus*	*Podocarpus macrophyllus*	WBG	KT030792	AY450866	JQ512476	693
		*Podocarpus neriifolius*	WBG	KT030793	AY450867	HM593765	693
		*Podocarpus totara*	–	NC_020361	NC_020361	NC_020361	683
		*Podocarpus lambertii*	–	KJ010812	KJ010812	KJ010812	683
	*Retrophyllum*	*Retrophyllum piresii*	–	NC_024827	NC_024827	NC_024827	683
	*Nageia*	*Nageia nagi*	WBG	KT030794	AY450868	AB644449	683
		*Nageia nagi*	–	NC_023120	NC_023120	NC_023120	683
		*Nageia fleuryi*	WBG	KT030795	HM593612	HM593714	683
Araucariaceae	*Araucaria*	*Araucaria cunninghamii*	WBG	KT030797	JQ512518	JQ512394	849
	*Agathis*	*Agathis dammara*	–	NC_023119	NC_023119	NC_023119	819
Taxaceae	*Taxus*	*Taxus media*	WBG	KT030780	EF660722	EF660664	753
		*Taxus wallichiana*	WBG	KT030784	EF660717	DQ478792	736
		*Taxus chinensis*	WBG	KT030782	AY450855	AF228103	759
		*Taxus cuspidata*	WBG	KT030781	EF660720	JX174691	759
		*Taxus yunnanensis*	IB	KT030783	AY450857	JX174720.1	767
		*Taxus mairei*	–	NC_020321	NC_020321	NC_020321	735
		*Taxus mairei*	–	KJ123824	KJ123824	KJ123824	773
	*Pseudotaxus*	*Pseudotaxus chienii*	SYSU	KT030788	AY450858	AF228105	657
	*Torreya*	*Torreya fargesii*	WBG	KT030785	EF660735	AF228107	871
		*Torreya grandis*	WBG	KT030786	EF660733	AF228108	879
	*Amentotaxus*	*Amentotaxus argotaenia*	IB	KT030787	AY450859	JQ512393	916
		*Amentotaxus formosana*	–	NC_024945	NC_024945	NC_024945	910
Cephalotaxaceae	*Cephalotaxus*	*Cephalotaxus hainanensis*	IB	KT030790	AY450862		1070
		*Cephalotaxus sinensis*	WBG	KT030789	AY450864	AB023988	1038
		*Cephalotaxus fortunei*	WBG	KT030791	AY450863	FJ600911	1038
		*Cephalotaxus wilsoniana*	–	NC_016063	NC_016063	NC_016063	1056
		*Cephalotaxus oliveri*	–	KC136217	KC136217	KC136217	936
Cupressaceae	*Taiwania*	*Taiwania cryptomerioides*	–	NC_016065	NC_016065	NC_016065	800
		*Taiwania flousiana*		NC_021441	NC_021441	NC_021441	795
	*Cryptomeria*	*Cryptomeria japonica*		NC_010548	NC_010548	NC_010548	700
	*Metasequoia*	*Metasequoia glyptostroboides*	WBG	KT030796	JQ512563	JQ512439	697
	*Calocedrus*	*Calocedrus formosana*		NC_023121	NC_023121	NC_023121	800
	*Cunninghamia*	*Cunninghamia lanceolata*		NC_021437	NC_021437	NC_021437	740
	*Juniperus*	*Juniperus virginiana*		KF866300.1	KF866300.1	KF866300.1	902
		*Juniperus monosperma*		KF866298	KF866298	KF866298	896
		*Juniperus scopulorum*		KF866299	KF866299	KF866299	884
		*Juniperus bermudiana*		KF866297	KF866297	KF866297	899


**WBG, Wuhan Botanical Garden, Chinese Academy of Sciences; IB, Institute of Botany, Chinese Academy of Sciences; SYSU, Sun Yat-sen University. The underlined accession numbers denote sequences experimentally determined in the present study*.*

### DNA Extraction and Sequencing

Total genomic DNA was isolated from the leaves of samples using the CTAB method (Gawel and Jarret, 1991). The quality of the genomic DNA was determined by 1% agarose gel electrophoresis. The *accD* gene investigated in this study was acquired using polymerase chain reaction (PCR). PCR primers (Supplementary Table 1) were designed from conserved region sequences in four gymnosperms (*C. japonica*, NC_010548; *T. cryptomerioides*, NC_016065; *C. wilsoniana*, NC_016063; *C. oliveri*, KC136217). The PCR system was as described in former study (Li et al., 2016). Then the PCR products were cloned into PCR 2.1 plasmid vector (Invitrogen, Carlsbad, CA, United States), and transformed to *E. coli* DH5α. At least three random positive clones were sequenced using ABI 3730xl DNA Analyzer (Applied Biosystems, Foster City, CA, United States).

### Sequence Assembly and Annotation

The sequences generated from different primers were assembled as a single sequence by BioEdit (Hall, 1999) with an overlapping of 150–300 bp. Contigs were initially annotated by DOGMA (Dual Organellar GenoMe Annotator). Genes that not be confirmed by DOGMA were recognized using Blastx^1^ and ORF Finder^2^. The tRNA genes were annotated by tRNAscan-SE v1.21 (Lowe and Eddy, 1997).

### Repeat Sequence Analyses

The sequences were initially scanned by REPuter at a repeat length ≥20 bp with a similarity of above 90% (Kurtz et al., 2001). Sequences were further processed by the Tandem Repeats Finder software (Benson, 1999).

### The Estimation of Substitution Rate

For the analysis in **Figure 2**, we first constructed a maximum likelihood (ML) tree using *rbcL* sequences. The analysis was performed in RaxML v8.1.x software with the GTR+I+G model. In addition, according to the strongly supported relationship published elsewhere (Lu et al., 2014), the Podocarpaceae and Araucariaceae were adjusted as a sister group for the *rbcL* ML tree. At last, this tree was used for the following substitution rate calculation. In order to compare the substitution rate of *accD* gene to two other widely used chloroplast gene marker *rbcL* and *matK*, we also downloaded these two gene sequences from GenBank. The branch lengths of non-synonymous (*d*_N_) and synonymous (*d*_S_) nucleotide substitutions for *accD*, *matK*, and *rbcL* trees were calculated using the free-ratio model implemented in PAML Codeml program.

## Results

### The General Features of accD Gene in Cupressophytes

The sequences acquired in this study were deposited in the GenBank with the accession number of KT30780-KT30797. A comparison of 57 gymnosperm *accD* sequences showed that the approximate 200 amino-acids at the end of this gene were highly conserved (Supplementary Figures 1-9, 1-10, the position of 1200–1400 in the alignment). This C-terminal region is functional importance for ACCD protein (Zhang et al., 2003). However, we found that the residues at the N-terminal and the middle region showed low similarities (Supplementary Figures 1-1 to 1-8). The major difference between 57 gymnosperm *accD* sequences is apparent as a large insertion sequences in the N-terminal and the middle region of cupressophyte *accD* sequence (Supplementary Figures 1-1 to 1-8). Furthermore, the open reading frame has not been destroyed by these insertion sequences.

The *accD* gene length in cupressophyte experienced an extraordinary expansion. The *accD* gene in Podocarpaceae lineage expands above 600 codons (**Table 1**). The *Cephalotaxus hainanensis* analyzed in this study shows the largest *accD* gene size, reaching 1070 codons (**Table 1**), which is approximately three times of the other Pinaceae species. The *accD* gene length also varies significantly within family. The *accD* gene in *Taxus* has experienced dramatic expansion, reaching as long as 735, 736, 753, 759, and 767 codons in *T. mairei*, *T. wallichiiana*, *T. media*, *T. chinensis*, and *T. yunnanensis*, respectively (**Table 1**); *P. chienii* has only 657 codons; *T. fargesii* and *T. grandis* have 871 codons and 879 codons, respectively; the *accD* gene length of *A. argotaenia* in Taxaceae is the longest, possessing 916 codons (**Table 1**). In general, our results support Hirao et al’s hypothesis that the *accD* gene length in cupressophytes has been expanded (Hirao et al., 2008).

### Repetitive Amino Acid Elements in *accD*

**FIGURE 1:**
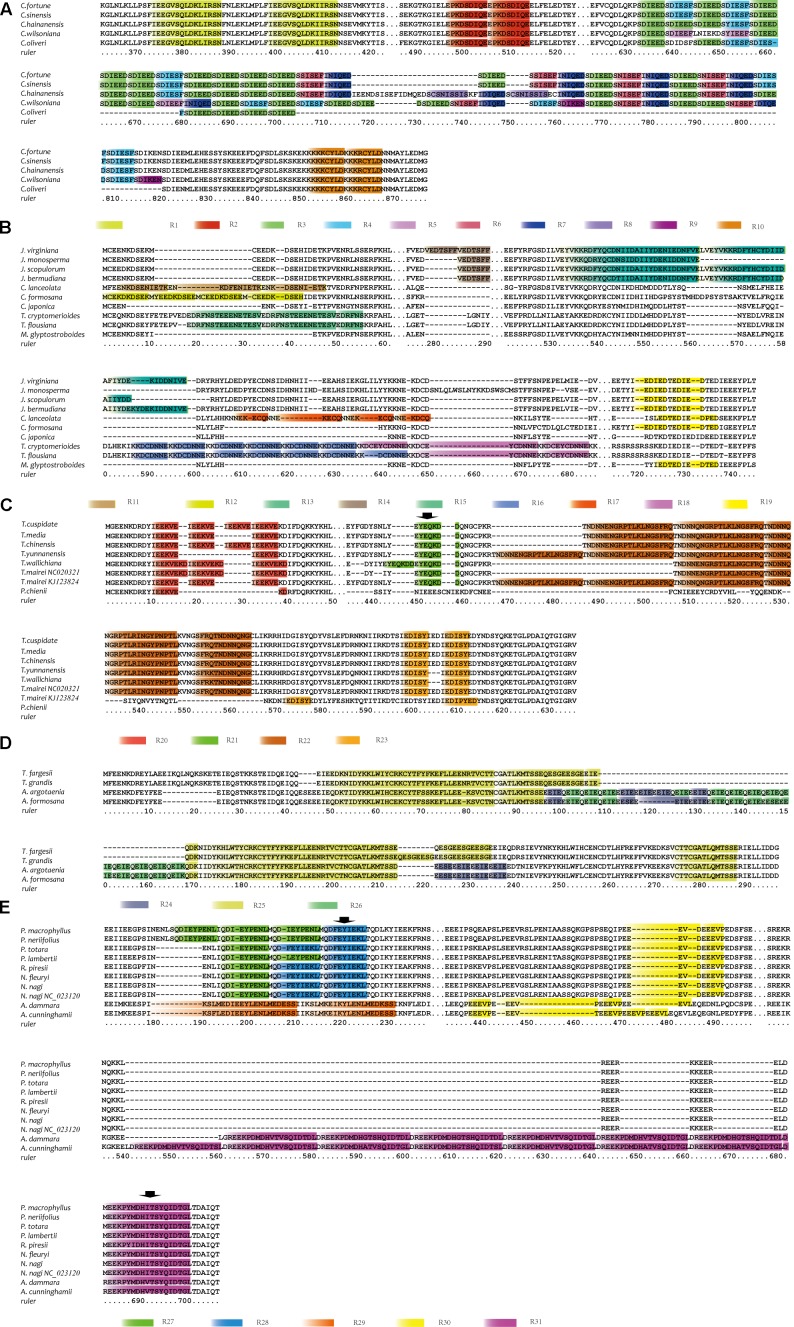
The repetitive elements of *accD* gene in five groups of species. Sequence alignments were performed individually in each group using MEGA. **(A)** The alignment of amino acid sequences of ACCD protein from five Cephalotaxaceae species. The figure only shows the regions from 364 to 422 and 488 to 876. **(B)** The alignment of amino acid sequences of ACCD protein from ten Cupressaceae species. The figure only shows the regions from 1 to 61, 276 to 290, and 522 to 744. **(C)** The alignment of amino acid sequences of ACCD protein from seven *Taxus* species and *P. chienii*. The figure only shows the regions from 1 to 50 and 436 to 636. **(D)** The alignment of amino acid sequences of ACCD protein from two *Torreya* and two *Amentotaxus* species. The figure only shows the regions from 1 to 297. **(E)** The alignment of amino acid sequences of ACCD protein from eight Podocarpaceae and two Araucariaceae species. The figure only shows the regions from 171 to 237, 434 to 497 and 530 to 707. Different repetitive elements were marked with different colored boxes. The arrows indicate the repetitive elements which has only one copy. The spacer between two fragments was divided by three dots.

To initiate our investigation into the mechanisms underlying *accD* gene length-associated mutation, REPuter and Tandem Repeat Finder were used to search repetitive sequences. As expected, *accD* gene length variation is explained by the insertions consisting of tandem repeated sequences. The repetitive sequences in *accD* gene are represented by a total of 31 categories present in 2–13 nearly identical copies, all of which are in the same (i.e., direct) orientation relative to each other (Supplementary Table 2 and **Figure 1**). Cycadaceae, Ginkgoaceae and the Pinaceae species with a relatively small gene size (**Table 1**) do not have repetitive elements. In comparison, the *accD* in cupressophytes investigated in this study possess a great many repetitive sequences. Ten repetitive elements were identified in the *accD* gene from the Cephalotaxaceae (Supplementary Table 2 and **Figure 1A**). Some repetitive elements, represented by R5, R9, and R8, were exclusively found either in *C. wilsoniana* or *C. hainanensis*, whereas the other repetitive elements such as R1, R2, R3, R4, R10 were found in all *Cephalotaxus* species. R1, R2, and R10 repetitive elements were all duplicated two times in the five *Cephalotaxus* species. The copy number of R3, R4, R6, and R7 varies in different species. For instance, the *C. hainanensis* has 13 repetitive elements of R3; while *C. sinensis*, *C. Wilsoniana*, and *C. fortune* have 12 copies of this repetitive element and *C. oliveri* has only six copies of R3 repetitive elements.

Three repetitive elements of R11 and four of R12 were found in *Cunninghamia lanceolata* and *Calocedrus formosana*, respectively (Supplementary Table 2 and **Figure 1B**). *Juniperus virginiana* has two copies of R14 while the other *Juniperus* species have only one copy of this repetitive element (Supplementary Table 2 and **Figure 1B**). The main difference in repetitive elements between two *Taiwania* species was the copy number variations of R16 and R18. *T. cryptomerioides* has six copies of R16 and three copies of R18, while *T. flousiana* has seven copies of R16 and two copies of R18 (Supplementary Table 2 and **Figure 1B**). R19 repetitive element commonly exists in Cupressaceae species except for *C. lanceolata* and *Taiwania* (Supplementary Table 2 and **Figure 1B**).

In *Taxus* and *Pseudotaxus*, the *accD* gene contained four kinds of repetitive elements: R20, R21, R22 and R23 (Supplementary Table 2 and **Figure 1C**). Two copies of R21 were found in *T. wallichiana* while other *Taxus* species have only one copy of R21. *T. chinensis* and *T. cuspidate* both have four copies of R20 and *T. yunnanensis* has only two, while the remaining four *Taxu*s species each have three copies of this repetitive elements. *P. chienii* also has R20 element but only one copy. The copy number of R22 in *Taxus* is also different, ranging from two in *T. wallichiana* to four in *T. yunnanensis.*

The largest tandem repetitive elements, spanning 59 amino acids, named as R25, exist in *Torreya* and *Amentotaxus* (Supplementary Table 2 and **Figure 1D**). Two copies of R25 were identified in *Torreya* and *Amentotaxus. Amentotaxus* has two genus-specific repetitive elements, R24 and R26, whose copy number are also different between *Amentotaxus argotaenia* and *Amentotaxus formosana* (Supplementary Table 2 and **Figure 1D**). Podocarpaceae has only a few repetitive elements (Supplementary Table 2 and **Figure 1E**). *P. macrophyllus* and *P. neriifolius* each contain three copies of R27, while *P. lambertii* contains two. Other *Podocarpaceae* species contain two copies of R28. *A. cunninghamii and A. dammara* each have eight and seven copies of R31, which is also lineage specific (Supplementary Table 2 and **Figure 1E**). The consensus sequences of R31 were also found in Podocarpaceae but all existing as single copy (not repeated), suggesting that R31 repetitive element was only duplicated in Araucariaceae. Furthermore, no pairs of direct repetitive sequences were identified in two sides of the inserted repetitive elements of cupressophytes.

### Rapid Evolution of *accD* in Cupressophytes

**FIGURE 2:**
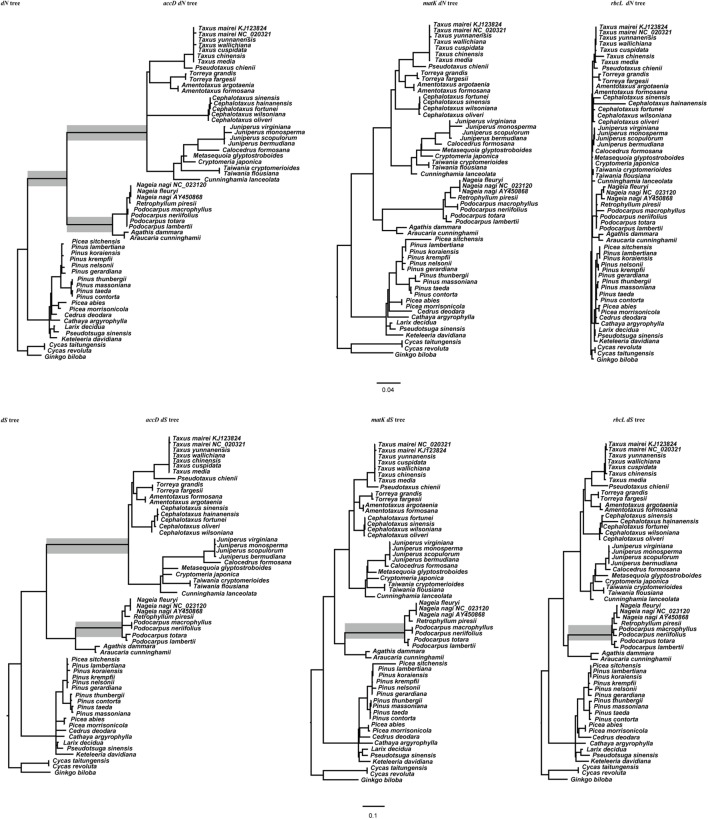
*d*_N_ and *d*_S_ trees for *accD*, *matK*, and *rbcL* gene. Branch lengths are in terms of *d*_N_ and *d*_S_ as estimated by PAML under a constrained topology. The topology of the *accD d*_N_ tree, *accD d*_S_ tree, *rbcL d*_N_ tree, *rbcL d*_S_ tree were identical to each other. The *matK d*_N_ tree and *d*_S_ tree were similar with *rbcL* and *accD* trees after removing *C. hainanensis*. The gray boxes denote the branch whose *d*_N_ or *d*_S_ has been accelerated.

The value of *d*_N_ and *d*_S_ for *accD*, *rbcL*, and *matK* gene were represented as branch lengths in **Figure 2**. In the *d*_N_ tree, *rbcL* and *matK* gene has a relatively low substitute rate through the entire tree. The branch leading to the ancestry clade of Cupressaceae, Taxaceae, and Cephalotaxaceae in the *accD d*_N_ tree is longer than other branches, suggesting that *accD* evolves faster in this clade. In addition, the branch leading to Podocarpaceae and Araucariaceae in the *accD d*_N_ tree is longer than other gymnosperms (**Figure 2**). Interestingly, the *accD* gene length also starts to expand at the lineage of Podocarpaceae and Araucariaceae. For the *matK* and *rbcL d*_S_ tree, most gymnosperm species evolve slowly and consistently except for the branch leading to Podocarpaceae. However, the *d*_S_ value of *accD* gene for cupressophyte evolves much faster than many of the Pinaceae species. Compared with *rbcL* and *matK*, *accD* gene shows a high level of divergence among cupressophyte species. In general, *accD* has experienced substitution rates acceleration and this acceleration is locus and lineage specific.

### Gene Order Around *accD* in Gymnosperms

**FIGURE 3:**
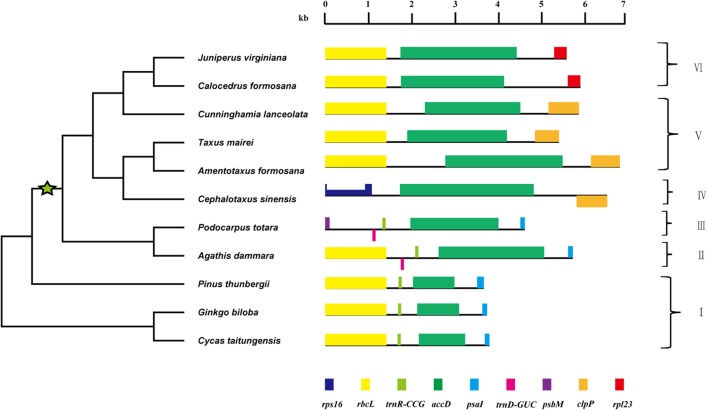
Gene organization around the *accD* locus in gymnosperms. Green star indicates the lineage where *accD* has expanded. Genes shown above line are transcribed from left to right, while those located below line are transcribed opposite direction. The half-height region in *rps16* represents an intron. The topology (not drawn to scale) in the left side was the same as *accD d*_N_ tree in **Figure 2**. The roman numbers I–VI denotes six types of gene organization around the *accD.*

The gene order around *accD* could be classified into six types (**Figure 3**). At high taxonomic levels, the gene order tends to be conserved across Cycadaceae, Ginkgoaceae and Pinaceae with a type of: *rbcL-trnR-accD-psaI*. Gene order in Araucariaceae and Podocarpaceae excluding *Podocarpus totara* is nearly identical to that of Cycadaceae, Ginkgoaceae and Pinaceae except that an extra *trnD* gene was found between *rbcL* and *trnR*. In *P. totara*, the gene order is: *psbM-trnD-accD-psaI*, which is different from that of the other three *Podocarpus* species, despite being members of the same genus. In Taxaceae, *C. japonica*, *Taiwania*, *M. glyptostroboides* and *C. lanceolata*, *rbcL* and *clpP* is near *accD*. The gene order of *Cephalotaxus* differs from that of Taxaceae by the inversion of *clpP* and translocation of *rps16*. Comparing with Taxaceae, the *rpl23* takes the place of *clpP* making the gene order to be: *rbcL-accD-rpl23* in *Juniperus* and *C. formosana*. It is amazing that gymnosperm chloroplast genomes have so much difference in gene organization surrounding *accD*, so we speculate that the *accD* gene must be involved in some rearrangement events of gymnosperm chloroplast genome.

## Discussion

### The *accD* Gene Length and Repetitive Elements

In gymnosperms, the reading frame lengths of *accD* vary considerably. At present, six complete chloroplast (cp) genomes of Gnetales have been published. However, *accD* could not be found in these cp genomes, suggesting that *accD* was lost from the cp genomes of Gnetales (Wu et al., 2009). The *accD* gene length of Cycas (359 codons) and Ginkgo (323 codons) is relatively short. In Pinaceae, the *accD* gene length range from 319 (*Picea abies*) to 326 (*Picea morrisonicola*) codons. However, we identified that the *accD* gene length in cupressophyte experienced an extraordinary expansion. From the alignment of 57 gymnosperm *accD* gene sequences, we can speculate that the enlarged *accD* gene size in cupressophytes is mainly caused by numerous amounts of insertion repetitive sequences in the middle region. Meanwhile, many different repetitive elements were identified in the inserted sequence. The repetitive elements have a relatively low similarity among different genus (Supplementary Table 2 and **Figure 1**), suggesting these repetitive elements likely do not have a common origin, and have formed independently.

### The Function of Repetitive Elements in *accD*

In addition to cupressophyte, the repeat sequences in *accD* were also reported in two legume species (*P. sativum* and *L. sativus*) (Magee et al., 2010), pepper (*C. annuum*) (Jo et al., 2011) and *M. truncatula* (Gurdon and Maliga, 2014). This verifies the idea that some proteins are more easily generating repeats during evolution (Mularoni et al., 2010). The *accD* gene in both *P. sativum* and *L. sativus* contains many repeat sequences in their middle region, but the repetitive elements from these two species show low similarity. The repetitive elements in *P. sativum* and *L. sativus* were also different with those in cupressophyte, suggesting that repetitive elements were species-specific. In pepper, seven repeats of an 18 bp-long element sequences were observed. And interestingly, one pair of short direct repeat sequences was located nearby the inserted repeat sequences. But no such sequences were found near the inserted repeat sequences of *accD* gene in cupressophytes and legume, suggesting that these direct repeat sequences were not necessary for the formation of repeat sequences. The transcription of *accD* gene in pepper was confirmed by reverse transcriptase PCR, so the expanded *accD* gene in pepper is supposed to be functional. Furthermore, a large number of complex repeats were found in the different ecotype of *M. truncatula*. It is suggested that the function of these inserted repeat sequences is not very important for ACCase (Gurdon and Maliga, 2014). However, on the other hand, the reading frame in this gene was not destroyed; so we speculate that the repetitive elements in these species may play a role of regulation to protein function. All of these results suggest that *accD* is a specific gene that tends to be easily form independently repeat sequence. And these repeat sequences are species-specific, which were only detected in some species.

The *accD* gene encodes the carboxyltransferase β subunit of ACCase. It is essential for leaf development in tobacco, as knocking out *accD* gene may be lethal (Kode et al., 2005). Three points strongly indicate that the function of this gene has not been destroyed in cupressophytes. Firstly, despite containing repeats, the original reading frame of *accD* gene is maintained, revealing that the genes in cupressophytes work well. Secondly, three sites were considered to be important for *accD* gene in potato: an acetyl-CoA bonding site, a CoA-carboxylation catalytic site and a carboxy-biotin binding site. All these three sites were located at the C-terminal region of the protein in all gymnosperm species. Thirdly, only the lineages of cupressophytes contain a large number of complex repeats. The Cycas (359 amino acids), Ginkgo (323 amino acids) (**Table 1**) and most angiosperm *accD* genes have not been expanded and did not contain repeat sequences.

Yi et al. (2013) have confirmed that *accD* in *C. oliveri* still have function after expansion. From the alignment of *accD* gene in 57 gymnosperms, we could see that the *accD* gene is considerably conserved in the 3′ end. In comparison, the nucleus copies of *accD* in *Trachelium* and *Trifolium* each encodes only the 3′ end region of the chloroplastic gene (Rousseau et al., 2013). The 3′ end region of the *accD* gene encode the carboxyl transferase, which is the most important functional region discovered in this gene (Zhang et al., 2003). So it is reasonable to see a higher conservable 3′ end of the *accD* relative to the much variable 5′ end as their function restriction.

### The Acceleration of Substitution Rate

Gene-specific rate acceleration is considered to be a common character of chloroplast genome evolution (Jansen et al., 2007; Park et al., 2017). Among three genes (*rbcL*, *matK*, *accD*) analyzed in this study, only *accD* gene shows an obviously acceleration in both *d*_N_ and *d*_S_ in cupressophyte (**Figure 2**), which was also the lineage full of abundant repeat sequences (Supplementary Table 2 and **Figure 1**). Meanwhile, the repetitive elements were also identified for *rbcL* and *matK* using Tandem Repeat Finder, but no repetitive elements were found. It seems that there is a positive correlation between repetitive elements and substitution rate acceleration. Many studies support that species-specific rate acceleration have relevant with genomic rearrangement (Jansen et al., 2007; Guisinger et al., 2008, 2010). However, the relationship between repeat sequence and rate acceleration has been little documented. Park et al. (2017) attribute the acceleration substitutate rate of *accD* gene in Geraniaceae to the insertion sequence. However, we found that repetitive elements also exist in these insertion sequences. Maybe the insertion of repetitive elements promoted the sequence to be more variable, thus leading to the acceleration of substitution rate.

### Genome Rearrangement Happened Near *accD*

Dispersed repeat elements were supposed to locate in rearrangement endpoints. In this study, we also sequenced the gene nearby *accD*, and found that there are six kinds of gene order type near *accD* (**Figure 3**). This indicates that *accD* gene is located in inversion endpoints. In addition to the relocation or complete loss of *trnD* or *trnR*, *rbcL* is generally located on one side of *accD*. For *Cephalotaxus*, a large inversion has happened relative to other gymnosperms which relocated *accD* gene near *rps16* rather than *rbcL* (Wu et al., 2011). We speculated that repetitive elements may induce the rearrangement near *accD*. An explanation for this correlation is that recombination between repeat sequence can lead to rearrangements of genome (Rogalski et al., 2006; Gray et al., 2009). In addition to *accD*, other rearrangement endpoints causing by inversions also exist. For instance, two inversions were identified between *Agathis dammara* and *Nageia nagi* chloroplast genome (Wu and Chaw, 2014), making the intergenic region of *ycf1* and *clpP*, *rpl23*, *rpl20*, and *petG* as rearrangement endpoints. However, repeat elements were only detected in the intergenic region of *ycf1* and *clpP. Rpl23*, *rpl20*, and *petG* did not have repeat sequence. These suggest that not all the rearrangement endpoints have repeat elements.

## Conclusion

The *accD* gene in cupressophyte has undergone an extraordinary length expansion, which was mainly caused by abundant independent repetitive elements. Accompanied with repetitive elements, the *d*_N_ and *d*_S_ of *accD* are also accelerated. In addition, *accD* has been involved in many rearrangement events. All these results suggest that the repetitive elements may promote the acceleration of substitution rate and mediate the genome rearrangement. Our study provides a typical case for the research of relationship between repetitive elements, genome rearrangement and substitution rate.

## Author Contributions

JL carried out the experiments, conducted data analysis, and wrote the manuscript. TW wrote the manuscript. YS designed the study and wrote the manuscript.

## Conflict of Interest Statement

The authors declare that the research was conducted in the absence of any commercial or financial relationships that could be construed as a potential conflict of interest. The handling Editor declared a shared affiliation, though no other collaboration, with one of the authors YS.

## Footnotes

1 http://blast.ncbi.nlm.nih.gov/Blast.cgi

2 https://www.ncbi.nlm.nih.gov/orffinder/

## References

[B1] Benson G. (1999). Tandem repeats finder: a program to analyze DNA sequences. *Nucleic Acids Res.* 27 573–580. 10.1093/nar/27.2.573 9862982 PMC148217

[B2] Bryant N. Lloyd J. Sweeney C. Myouga F. Meinke D. (2011). Identification of nuclear genes encoding chloroplast-localized proteins required for embryo development in Arabidopsis. *Plant Physiol.* 155 1678–1689. 10.1104/pp.110.168120 21139083 PMC3091104

[B3] Cai Z. Guisinger M. Kim H. G. Ruck E. Blazier J. C. McMurtry V. (2008). Extensive reorganization of the plastid genome of *Trifolium subterraneum* (Fabaceae) is associated with numerous repeated sequences and novel DNA insertions. *J. Mol. Evol.* 67 696–704. 10.1007/s00239-008-9180-7 19018585

[B4] Christenhusz M. J. M. Reveal J. L. Farjon A. Gardner M. F. Mill R. R. Chase M. W. (2011). A new classification and linear sequence of extant gymnosperms. *Phytotaxa* 19 55–70. 10.11646/phytotaxa.19.1.3

[B5] Chumley T. W. Palmer J. D. Mower J. P. Fourcade H. M. Calie P. J. Boore J. L. (2006). The complete chloroplast genome sequence of *Pelargonium* x *hortorum*: organization and evolution of the largest and most highly rearranged chloroplast genome of land plants. *Mol. Biol. Evol.* 23 2175–2190. 10.1093/molbev/msl089 16916942

[B6] Curci P. L. De Paola D. Danzi D. Vendramin G. G. Sonnante G. (2015). Complete chloroplast genome of the multifunctional crop globe artichoke and comparison with other Asteraceae. *PLoS One* 10:e0120589. 10.1371/journal.pone.0120589 25774672 PMC4361619

[B7] de Koning A. P. Keeling P. J. (2006). The complete plastid genome sequence of the parasitic green alga *Helicosporidium* sp. is highly reduced and structured. *BMC Biol.* 4:12. 10.1186/1741-7007-4-12 16630350 PMC1463013

[B8] Djian P. Hancock J. M. Chana H. S. (1996). Codon repeats in genes associated with human diseases: fewer repeats in the genes of nonhuman primates and nucleotide substitutions concentrated at the sites of reiteration. *Proc. Natl. Acad. Sci. U.S.A.* 93 417–421. 10.1073/pnas.93.1.417 8552651 PMC40249

[B9] Erixon P. Oxelman B. (2008). Whole-gene positive selection, elevated synonymous substitution rates, duplication, and indel evolution of the chloroplast *clpP1* gene. *PLoS One* 3:e1386. 10.1371/journal.pone.0001386 18167545 PMC2148103

[B10] Faux N. G. Huttley G. A. Mahmood K. Webb G. I. de la Banda M. G. Whisstock J. C. (2007). RCPdb: an evolutionary classification and codon usage database for repeat-containing proteins. *Genome. Res.* 17 1118–1127. 10.1101/gr.6255407 17567984 PMC1899123

[B11] Gawel N. Jarret R. (1991). A modified CTAB DNA extraction procedure for *musa* and *ipomoea*. *Plant Mol. Biol. Rep.* 9 262–266. 10.1007/bf02668371

[B12] Goremykin V. V. Holland B. Hirsch-Ernst K. I. Hellwig F. H. (2005). Analysis of *Acorus calamus* chloroplast genome and its phylogenetic implications. *Mol. Biol. Evol.* 22 1813–1822. 10.1093/molbev/msi173 15930156

[B13] Gornicki P. Faris J. King I. Podkowwinski J. Gill B. Haselkorn R. (1997). Plastid-localized acetyl-CoA carboxylase of bread wheat is encoded by a single gene on each of the three ancestral chromosome sets. *Proc. Natl. Acad. Sci. U.S.A.* 94 14179–14184. 10.1073/pnas.94.25.14179 9391173 PMC28453

[B14] Gray B. N. Ahner B. A. Hanson M. R. (2009). Extensive homologous recombination between introduced and native regulatory plastid DNA elements in transplastomic plants. *Transgenic Res.* 18 559–572. 10.1007/s11248-009-9246-3 19184502

[B15] Guisinger M. M. Chumley T. W. Kuehl J. V. Boore J. L. Jansen R. K. (2010). Implications of the plastid genome sequence of *Typha* (Typhaceae, Poales) for understanding genome evolution in Poaceae. *J. Mol. Evol.* 70 149–166. 10.1007/s00239-009-9317-3 20091301 PMC2825539

[B16] Guisinger M. M. Kuehl J. V. Boore J. L. Jansen R. K. (2008). Genome-wide analyses of Geraniaceae plastid DNA reveal unprecedented patterns of increased nucleotide substitutions. *Proc. Natl. Acad. Sci. U.S.A.* 105 18424–18429. 10.1073/pnas.0806759105 19011103 PMC2587588

[B17] Guisinger M. M. Kuehl J. V. Boore J. L. Jansen R. K. (2011). Extreme reconfiguration of plastid genomes in the angiosperm family Geraniaceae: rearrangements, repeats, and codon usage. *Mol. Biol. Evol.* 28 583–600. 10.1093/molbev/msq229 20805190

[B18] Guo X. Castillo-Ramirez S. Gonzalez V. Bustos P. Fernandez-Vazquez J. L. Santamaria R. I. (2007). Rapid evolutionary change of common bean (*Phaseolus vulgaris* L.) plastome, and the genomic diversification of legume chloroplasts. *BMC Genomics* 8:228. 10.1186/1471-2164-8-228 17623083 PMC1940014

[B19] Gurdon C. Maliga P. (2014). Two distinct plastid genome configurations and unprecedented intraspecies length variation in the *accD* coding region in *Medicago truncatula*. *DNA Res.* 21 417–427. 10.1093/dnares/dsu007 24644300 PMC4131835

[B20] Haberle R. C. Fourcade H. M. Boore J. L. Jansen R. K. (2008). Extensive rearrangements in the chloroplast genome of *Trachelium caeruleum* are associated with repeats and tRNA genes. *J. Mol. Evol.* 66 350–361. 10.1007/s00239-008-9086-4 18330485

[B21] Hall T. A. (1999). BioEdit:a user friendly biological sequence alignment editor and analysis program for windows 95/98/NT. *Nucleic Acids Symp. Ser.* 41 95–98.

[B22] Harris M. E. Meyer G. Vandergon T. Vandergon V. O. (2012). Loss of the acetyl-CoA carboxylase (*accD*) gene in Poales. *Plant Mol. Biol. Rep.* 31 21–31. 10.1007/s11105-012-0461-3

[B23] Hipkins V. D. Marshall K. A. Neale D. B. Rottmann W. H. Strauss S. H. (1995). A mutation hotspot in the chloroplast genome of a conifer (Douglas-fir: *Pseudotsuga*) is caused by variability in the number of direct repeats derived from a partially duplicated tRNA gene. *Curr. Genet.* 27 572–579. 10.1007/bf00314450 7553944

[B24] Hirao T. Watanabe A. Kurita M. Kondo T. Takata K. (2008). Complete nucleotide sequence of the *Cryptomeria japonica* D. Don. chloroplast genome and comparative chloroplast genomics: diversified genomic structure of coniferous species. *BMC Plant Biol.* 8:70. 10.1186/1471-2229-8-70 18570682 PMC2443145

[B25] Huntley M. Golding G. B. (2000). Evolution of simple sequence in proteins. *J. Mol. Evol.* 51 131–140. 10.1007/s002390010073 10948269

[B26] Huntley M. A. Clark A. G. (2007). Evolutionary analysis of amino acid repeats across the genomes of 12 Drosophila species. *Mol. Biol. Evol.* 24 2598–2609. 10.1093/molbev/msm129 17602168

[B27] Jansen R. K. Cai Z. Raubeson L. A. Daniell H. Depamphilis C. W. Leebens-Mack J. (2007). Analysis of 81 genes from 64 plastid genomes resolves relationships in angiosperms and identifies genome-scale evolutionary patterns. *Proc. Natl. Acad. Sci. U.S.A.* 104 19369–19374. 10.1073/pnas.0709121104 18048330 PMC2148296

[B28] Jo Y. D. Park J. Kim J. Song W. Hur C. G. Lee Y. H. (2011). Complete sequencing and comparative analyses of the pepper (*Capsicum annuum* L.) plastome revealed high frequency of tandem repeats and large insertion/deletions on pepper plastome. *Plant Cell Rep.* 30 217–229. 10.1007/s00299-010-0929-2 20978766

[B29] Kim K. J. Choi K. S. Jansen R. K. (2005). Two chloroplast DNA inversions originated simultaneously during the early evolution of the sunflower family (Asteraceae). *Mol. Biol. Evol.* 22 1783–1792. 10.1093/molbev/msi174 15917497

[B30] Kode V. Mudd E. A. Iamtham S. Day A. (2005). The tobacco plastid *accD* gene is essential and is required for leaf development. *Plant J.* 44 237–244. 10.1111/j.1365-313x.2005.02533.x 16212603

[B31] Konishi T. Sasaki Y. (1994). Compartmentalization of two forms of acetyl-CoA carboxylase in plants and the origin of their tolerance toward herbicides. *Proc. Natl. Acad. Sci. U.S.A.* 91 3598–3601. 10.1007/978-94-015-8394-7_15 7909603 PMC43627

[B32] Konishi T. Shinohara K. Yamada K. Sasaki Y. (1996). Acetyl-CoA carboxylase in higher plants: most plants other than *Gramineae* have both the prokaryotic and the eukaryotic forms of this enzyme. *Plant Cell Physiol.* 37:117–122. 10.1093/oxfordjournals.pcp.a028920 8665091

[B33] Kurtz S. Choudhuri J. Ohlebusch E. Schleiermacher C. Stoye J. Robert G. (2001). REPuter: the manifold applications of repeat analysis on a genomic scale. *Nucleic Acids Res.* 29 4633–4642. 10.1093/nar/29.22.4633 11713313 PMC92531

[B34] Lee H. L. Jansen R. K. Chumley T. W. Kim K. J. (2007). Gene relocations within chloroplast genomes of *Jasminum* and *Menodora* (Oleaceae) are due to multiple, overlapping inversions. *Mol. Biol. Evol.* 24 1161–1180. 10.1093/molbev/msm036 17329229

[B35] Li J. Gao L. Chen S. S. Tao K. Su Y. J. Wang T. (2016). Evolution of short inverted repeat in cupressophytes, transfer of *accD* to nucleus in *Sciadopitys verticillata* and phylogenetic position of Sciadopityaceae. *Sci. Rep.* 6:20934. 10.1038/srep20934 26865528 PMC4750060

[B36] Lowe T. M. Eddy S. R. (1997). tRNAscan-SE: a program for improved detection of transfer RNA genes in genomic sequence. *Nucleic Acids Res.* 25 955–964. 10.1093/nar/25.5.955 9023104 PMC146525

[B37] Lu Y. Ran J. H. Guo D. M. Yang Z. Y. Wang X. Q. (2014). Phylogeny and divergence times of gymnosperms inferred from single-copy nuclear genes. *PLoS One* 9:e107679. 10.1371/journal.pone.0107679 25222863 PMC4164646

[B38] Madoka Y. Tomizawa K. I. Mizoi J. Nishida I. Nagano Y. Sasaki Y. (2002). Chloroplast transformation with modified *accD* operon increases acetyl-CoA carboxylase and causes extension of leaf longevity and increase in seed yield in tobacco. *Plant Cell Physiol.* 43 1518–1525. 10.1093/pcp/pcf172 12514249

[B39] Magee A. M. Aspinall S. Rice D. W. Cusack B. P. Semon M. Perry A. S. (2010). Localized hypermutation and associated gene losses in legume chloroplast genomes. *Genome Res.* 20 1700–1710. 10.1101/gr.111955.110 20978141 PMC2989996

[B40] Mularoni L. Ledda A. Toll-Riera M. Alba M. M. (2010). Natural selection drives the accumulation of amino acid tandem repeats in human proteins. *Genome Res.* 20 745–754. 10.1101/gr.101261.109 20335526 PMC2877571

[B41] Park S. Ruhlman T. A. Weng M. L. Hajrah N. H. Sabir J. S. M. Jansen R. K. (2017). Contrasting patterns of nucleotide substitution rates provide insight into dynamic evolution of plastid and mitochondrial genomes of *Geranium*. *Genome Biol. Evol.* 9 1766–1780. 10.1093/gbe/evx124 28854633 PMC5570028

[B42] Rogalski M. Ruf S. Bock R. (2006). Tobacco plastid ribosomal protein S18 is essential for cell survival. *Nucleic Acids Res.* 34 4537–4545. 10.1093/nar/gkl634 16945948 PMC1636375

[B43] Rousseau G. M. Huang X. Higginson E. Ayliffe M. Day A. Timmis J. N. (2013). Potential functional replacement of the plastidic acetyl-CoA carboxylase subunit (*accD*) gene by recent transfers to the nucleus in some angiosperm lineages. *Plant Physiol.* 161 1918–1929. 10.1104/pp.113.214528 23435694 PMC3613465

[B44] Saski C. Lee S. B. Daniell H. Wood T. C. Tomkins J. Kim H. G. (2005). Complete chloroplast genome sequence of *Glycine max* and comparative analyses with other legume genomes. *Plant Mol. Biol.* 59 309–322. 10.1007/s11103-005-8882-0 16247559

[B45] Sasaki Y. Nagano Y. (2004). Plant acetyl-CoA carboxylase: structure, biosynthesis, regulation, and gene manipulation for plant breeding. *Biosci. Biotechnol. Biochem.* 68 1175–1184. 10.1271/bbb.68.1175 15215578

[B46] Simon M. Hancock J. M. (2009). Tandem and cryptic amino acid repeats accumulate in disordered regions of proteins. *Genome Biol.* 10:R59. 10.1186/gb-2009-10-6-r59 19486509 PMC2718493

[B47] Tangphatsornruang S. Uthaipaisanwong P. Sangsrakru D. Chanprasert J. Yoocha T. Jomchai N. (2011). Characterization of the complete chloroplast genome of *Hevea brasiliensis* reveals genome rearrangement, RNA editing sites and phylogenetic relationships. *Gene* 475 104–112. 10.1016/j.gene.2011.01.002 21241787

[B48] Timme R. E. Kuehl J. V. Boore J. L. Jansen R. K. (2007). A comparative analysis of the *Lactuca* and *Helianthus* (Asteraceae) plastid genomes: identifcation of divergent regions and categorazation of shared repeats. *Am. J. Bot.* 94 302–312. 10.3732/ajb.94.3.302 21636403

[B49] Tsai C. H. Strauss S. H. (1989). Dispersed repetitive sequences in the chloroplast genome of Douglas-fir. *Curr. Genet.* 16 211–218. 10.1007/bf00391479 2598277

[B50] Vieira L. N. Faoro H. Rogalski M. Fraga H. P. Cardoso R. L. (2014). The complete chloroplast genome sequence of *Podocarpus lambertii*: genome structure, evolutionary aspects, gene content and SSR detection. *PLoS One* 9:e90618. 10.1371/journal.pone.0090618 24594889 PMC3942463

[B51] Wolfe K. H. Mordent C. W. Palmers J. D. (1992). Function and evolution of a minimal plastid genome from a nonphotosynthetic parasitic plant. *Proc. Natl. Acad. Sci. U.S.A.* 89 10648–10652. 10.1073/pnas.89.22.10648 1332054 PMC50398

[B52] Wu C. S. Chaw S. M. (2014). Highly rearranged and size-variable chloroplast genomes in conifers II clade (cupressophytes): evolution towards shorter intergenic spacers. *Plant Biotechnol. J.* 12 344–353. 10.1111/pbi.12141 24283260

[B53] Wu C. S. Lai Y. T. Lin C. P. Wang Y. N. Chaw S. M. (2009). Evolution of reduced and compact chloroplast genomes (cpDNAs) in gnetophytes: selection toward a lower-cost strategy. *Mol. Phylogenet Evol.* 52 115–124. 10.1016/j.ympev.2008.12.026 19166950

[B54] Wu C. S. Wang Y. N. Hsu C. Y. Lin C. P. Chaw S. M. (2011). Loss of different inverted repeat copies from the chloroplast genomes of Pinaceae and cupressophytes and influence of heterotachy on the evaluation of gymnosperm phylogeny. *Genome Biol. Evol.* 3 1284–1295. 10.1093/gbe/evr095 21933779 PMC3219958

[B55] Yi X. Gao L. Wang B. Su Y. J. Wang T. (2013). The complete chloroplast genome sequence of *Cephalotaxus oliveri* (Cephalotaxaceae): evolutionary comparison of *Cephalotaxus* chloroplast DNAs and insights into the loss of inverted repeat copies in gymnosperms. *Genome Biol. Evol.* 5 688–698. 10.1093/gbe/evt042 23538991 PMC3641632

[B56] Zhang H. Yang Z. Shen Y. Tong L. (2003). Crystal structure of the carboxyltransferase domain of acetyl-coenzyme A carboxylase. *Science* 299 2064–2067. 10.2210/pdb3k8x/pdb 12663926

[B57] Zhang Y. Ma J. Yang B. Li R. Zhu W. Sun L. (2014). The complete chloroplast genome sequence of *Taxus chinensis* var. *mairei* (Taxaceae): loss of an inverted repeat region and comparative analysis with related species. *Gene* 540 201–209. 10.1016/j.gene.2014.02.037 24583178

[B58] Zhao Y. Yin J. Guo H. Zhang Y. Xiao W. Sun C. (2014). The complete chloroplast genome provides insight into the evolution and polymorphism of *Panax ginseng*. *Front. Plant Sci.* 5:696. 10.3389/fpls.2014.00696 25642231 PMC4294130

