# Supplementary material for: The Repeat Sequences and Elevated Substitution Rates of the Chloroplast accD Gene in Cupressophytes
Source: Front Plant Sci. 2018 Apr 20;9:533. doi: 10.3389/fpls.2018.00533 (PMC5920036; doi:10.3389/fpls.2018.00533)
Supplement: Supplementary file 1 [file Table_1.DOCX]

Supplementary Table 1

Primers used in this study.

| Primer name | location | direction | Primer Sequence (5'-3') |
| --- | --- | --- | --- |
| 1 | *rbcL*-*accD* | F | AGTCTCAATGTAATGCCAATCCAAC |
|  | *accD* | R | CTCTTTTACCTGCAAATGCAATGTA |
| 2 | *accD* | F | GTATGGGCTCGGTAGTGGGTG |
|  | *clpP* | R | CATCCCCTGTTACGTATTGCAT |
| 3 | *accD* | F | GTATGGGCTCGGTAGTGGGTG |
|  | *clpP* | R | TTCGAAATTGAATGGTTCGGAT |
| 4 | *psbK* | F | ACGCGAAAGAGATTTTACTGCT |
|  | *accD* | R | TTAAATTATCGCAAAACACAACC |
| 5 | *accD* | F | CAACATCCATCATATATCTTTCGC |
|  | *accD* | R | TAAAAGAGCTCGTGGAACCAT |
| 6 | *clpP* | F | CATCCCCTGTTACGTATTGCAT |
|  | *rpl33* | R | ATCGCCAGAATACAACTAGTCG |
| 7 | *rbcL-trnR* | F | CGATCCAGATTGAGATTTAC |
|  | *accD* | R | TTCTCACCYACTACMGAGCCCATAC |
| 8 | *accD* | F | ACAGTATACTCCAGTTTCAGCT |
|  | *accD* | R | CTCTTTTACCTGCAAATGCAATGTA |
| 9 | *accD* | F | GTATGGGCTCGGTAGTGGGTG |
|  | *ycf4* | R | GGTAACTTGAAATTCCGACCA |
| 10 | *rbcL* | F | TGCACTTCCGTGTACTGGCTA |
|  | *accD* | R | TTCTCACCYACTACMGAGCCCATAC |
| 11 | *accD* | F | GTATGGGCTCGGTAGTGGGTG |
|  | *cemA* | R | TATTCCACCAATTTGTAACC |
| 12 | *rbcL* | F | AAATGGAGTCCCGAACTAGCTG |
|  | *accD* | R | CTCTTTTACCTGCAAATGCAATGTA |
| 13 | *accD* | F | TAAAAGAGCTCGTGGAACCAT |
|  | *accD* | R | TACAATTCGGAACGTTTTAAGCA |
| 14 | *rbcL* | F | AAATGGAGTCCCGAACTAGCTG |
|  | *accD* | R | TTCTCACCYACTACMGAGCCCATAC |
| 15 | *accD* | F | ATTACGCCTTGTGAAGACCCT |
|  | *accD* | R | CTCTTTTACCTGCAAATGCAATGTA |
| 16 | *accD* | F | GTATGGGCTCGGTAGTGGGTG |
|  | *cemA* | R | TATTCCACCAATTTGTAACC |

Primers 1 and 2 were used to amplify *T. media*, *T. wallichiana*, *T. chinensis*, *T. cuspidata*, *T. yunnanensis*, *P. chienii* and *A. argotaenia*; Primers 1 and 3 were used to amplify *T. fargesii*, *T. grandis*; Primers 3, 4, 5, 6 for *C. hainanensis*, *C. sinensis*, *C. fortunei*; Primers 7, 8, 9 for *P. macrophyllus*; Primers 8, 10, 11 for *P. neriifolius*, *N. nagi*, *N. fleuryi*; Primers 2, 12, 13 for *M. glyptostroboides*; Primers 14,15,16 for *A. cunninghamii*.
