# Supplementary material for: The Repeat Sequences and Elevated Substitution Rates of the Chloroplast accD Gene in Cupressophytes
Source: Front Plant Sci. 2018 Apr 20;9:533. doi: 10.3389/fpls.2018.00533 (PMC5920036; doi:10.3389/fpls.2018.00533)
Supplement: Supplementary file 3 [file Presentation_1.PDF]

Supplementary Material

The Repeat Sequences and Elevated Substitution Rates of the Chloroplast *accD* Gene in Cupressophytes

Jia Li[1], Yingjuan Su[2,3], Ting Wang[4*]

*** Correspondence:** Corresponding Author: tingwang@scau.edu.cn

1 Supplementary Figures and Tables

1.1 Supplementary Figures

Supplementary Figure 1. Comparison of amino acid sequences of *accD* from 57 gymnosperms. The histogram below the sequences denotes the degree of similarity. Peaks and valleys represent sites of high and low similarity, respectively. Numbers on the bottom of the sequences indicate the relative position of the alignment sequences.

1.2 Supplementary Table

Supplementary table 1. Primers used in this study.

Supplementary table 2. Characterization of identified repetitive elements in *accD* sequences.
