# Supplementary material for: The Repeat Sequences and Elevated Substitution Rates of the Chloroplast accD Gene in Cupressophytes
Source: Front Plant Sci. 2018 Apr 20;9:533. doi: 10.3389/fpls.2018.00533 (PMC5920036; doi:10.3389/fpls.2018.00533)

Supplementary Figure 1-1

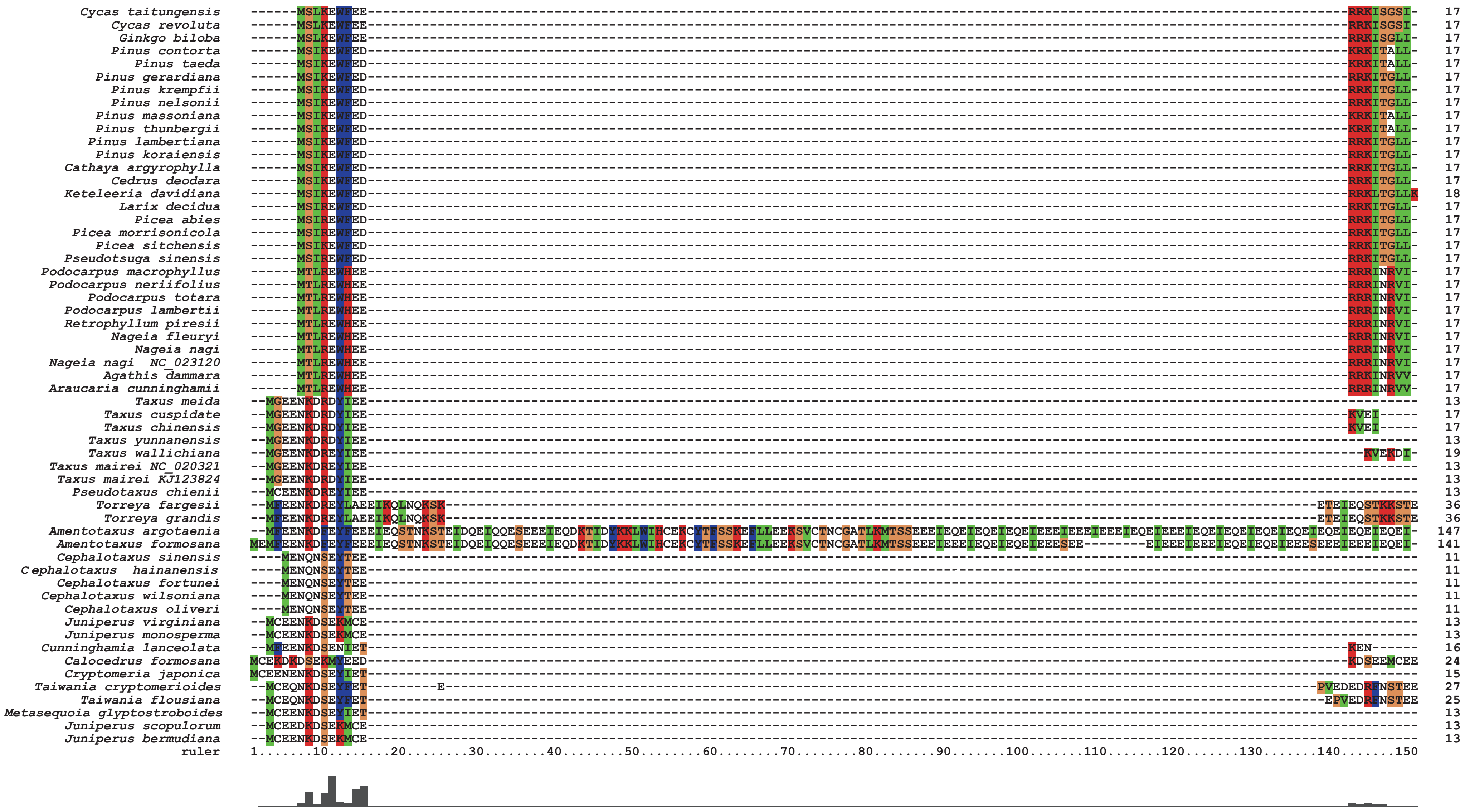

Supplementary Figure 1-2

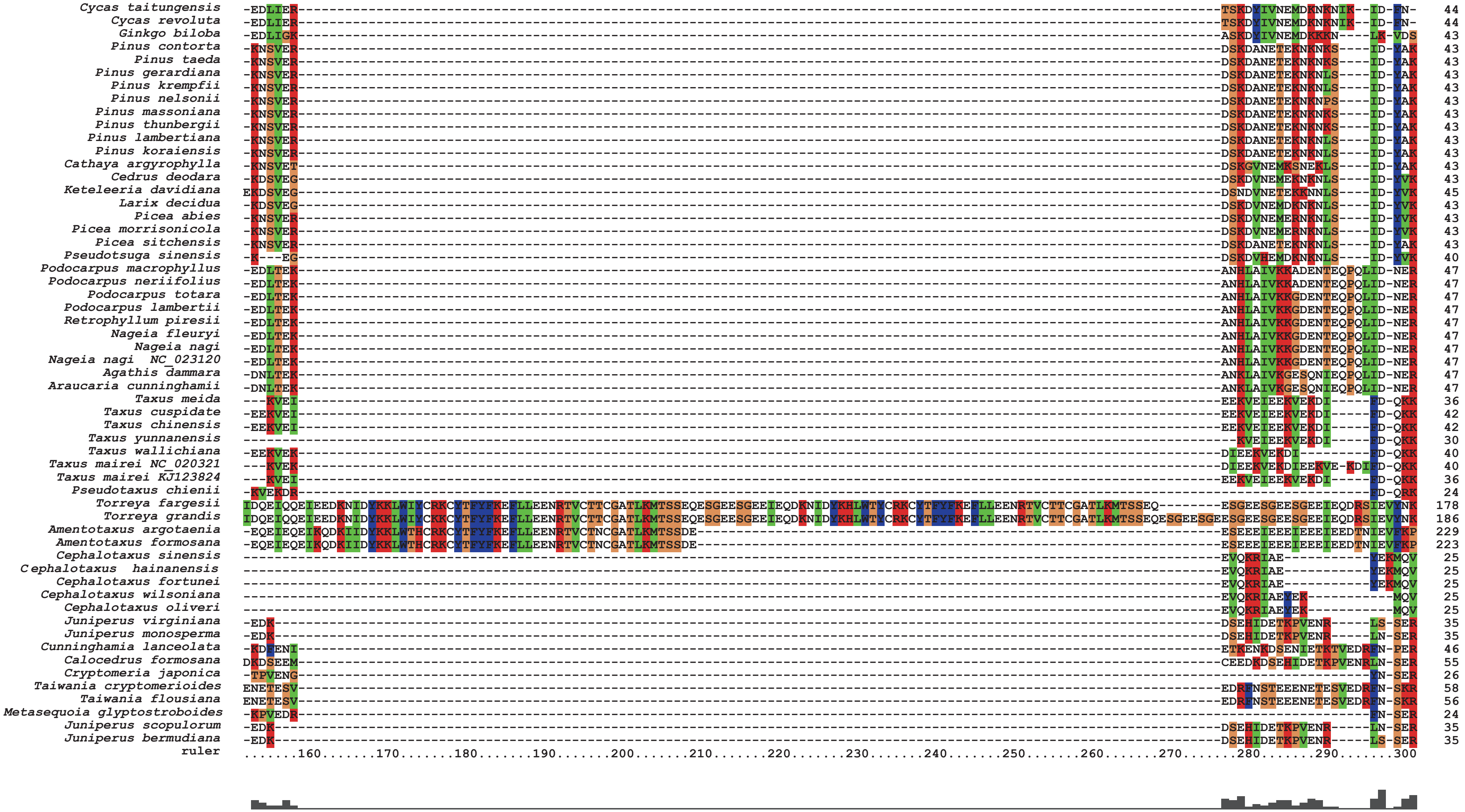

Supplementary Figure 1-3

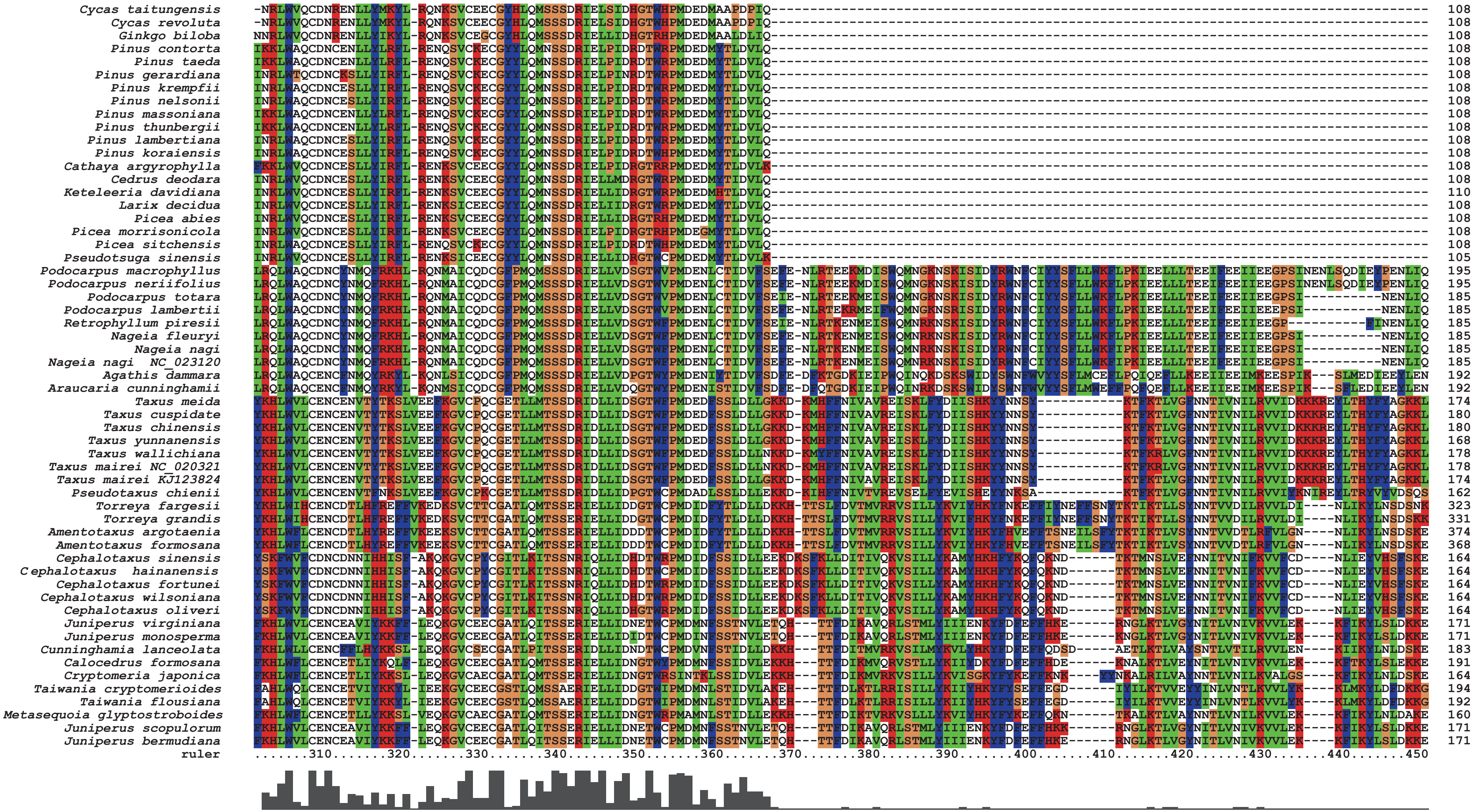

Supplementary Figure 1-4

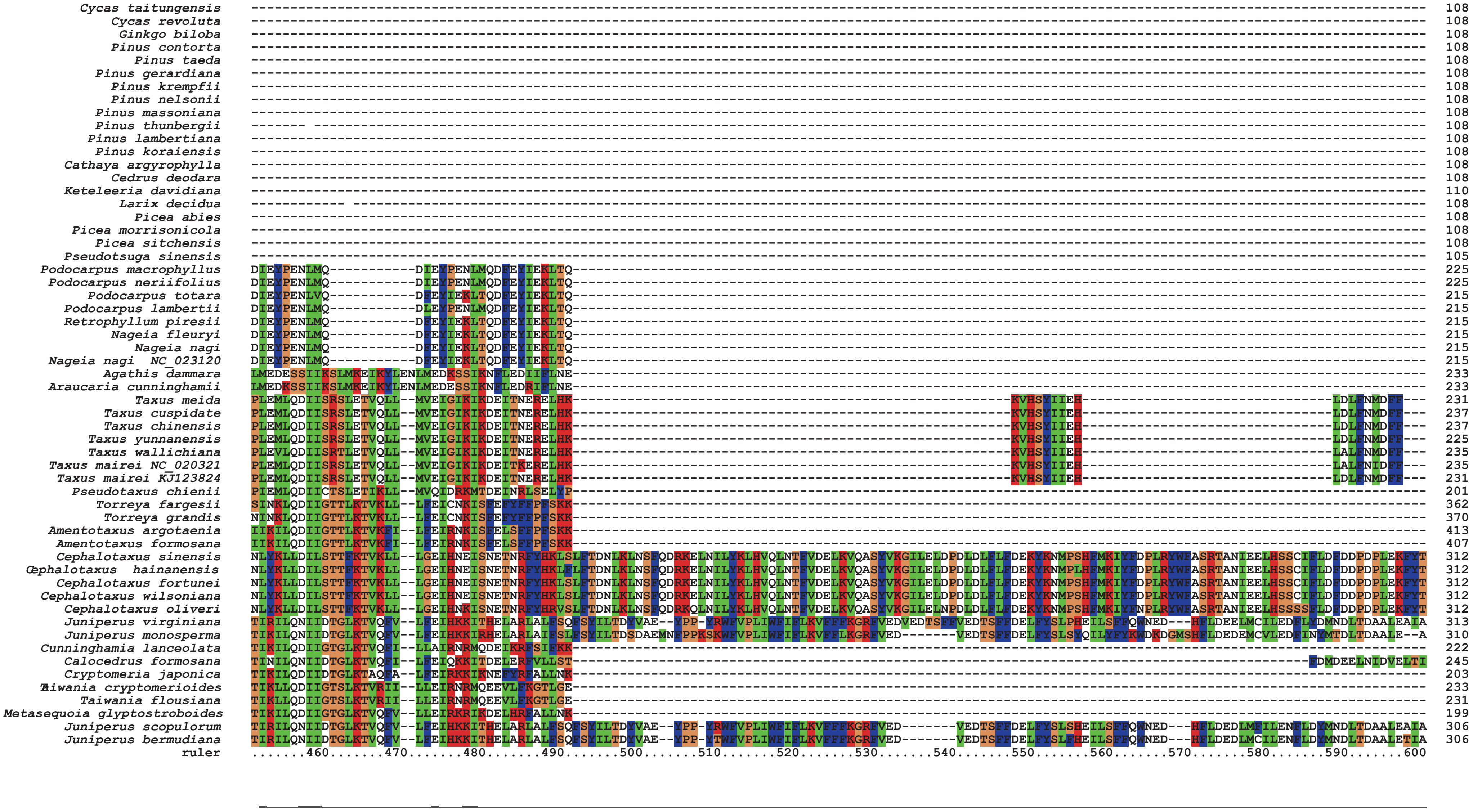

Supplementary Figure 1-5

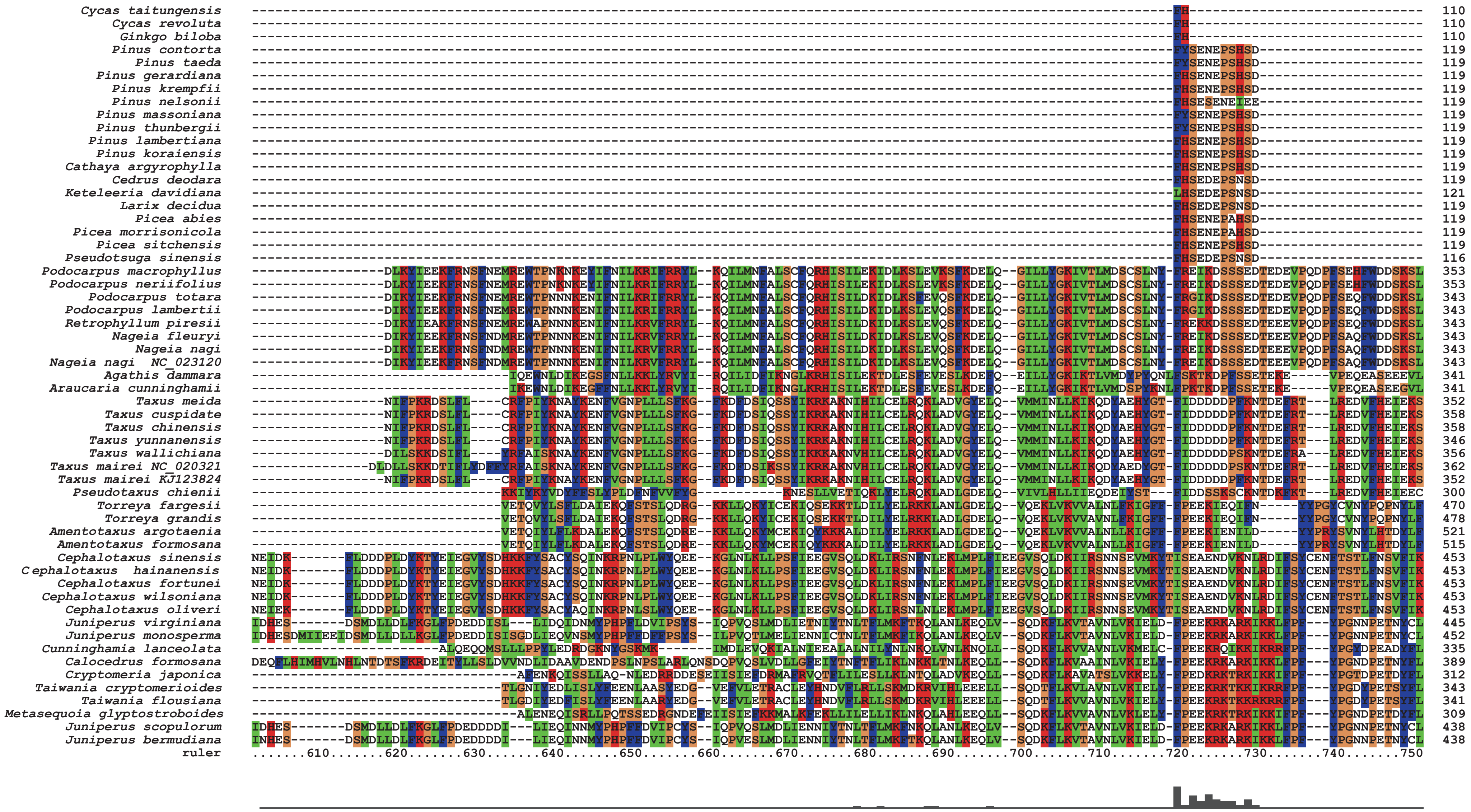

Supplementary Figure 1-6

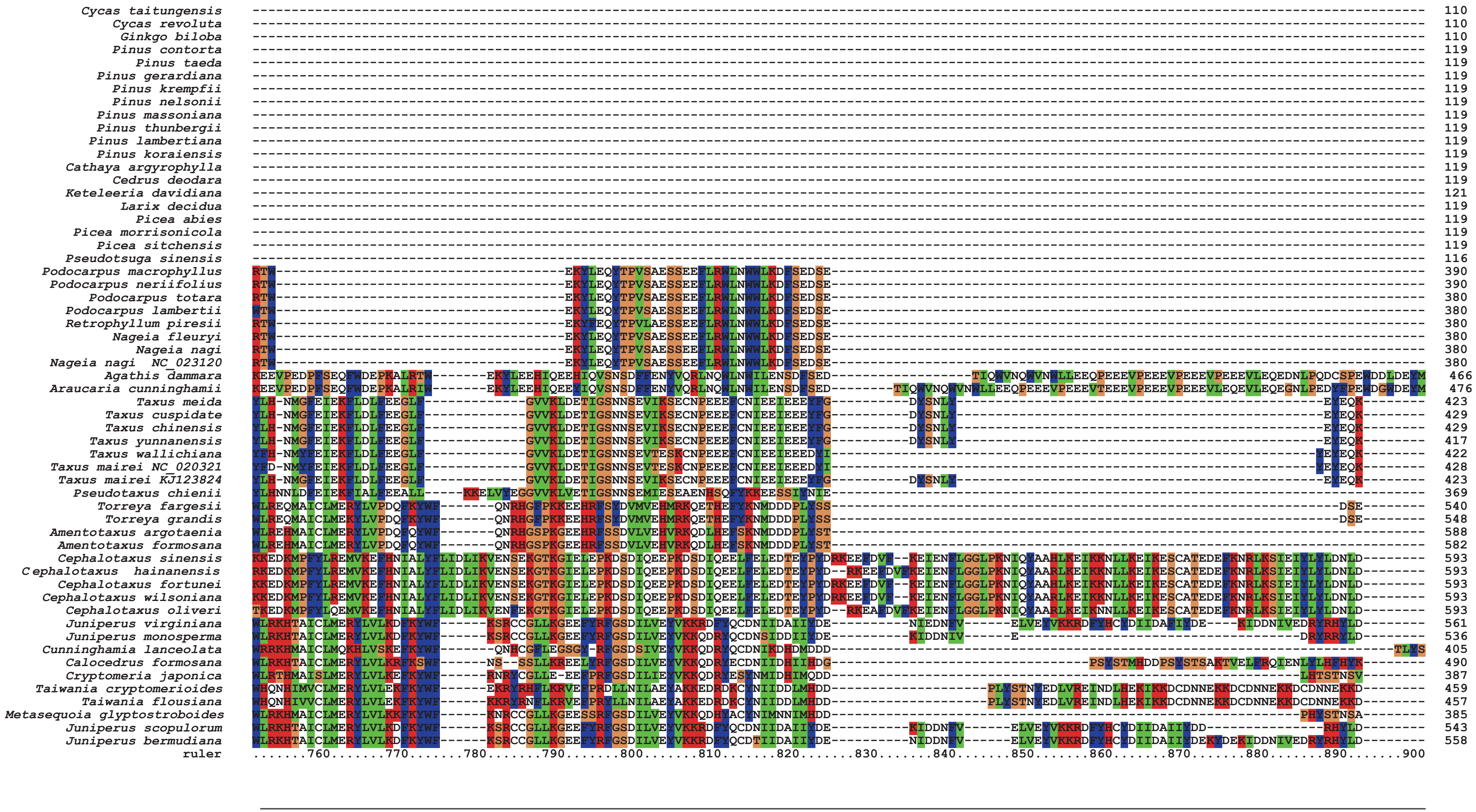

Supplementary Figure 1-7

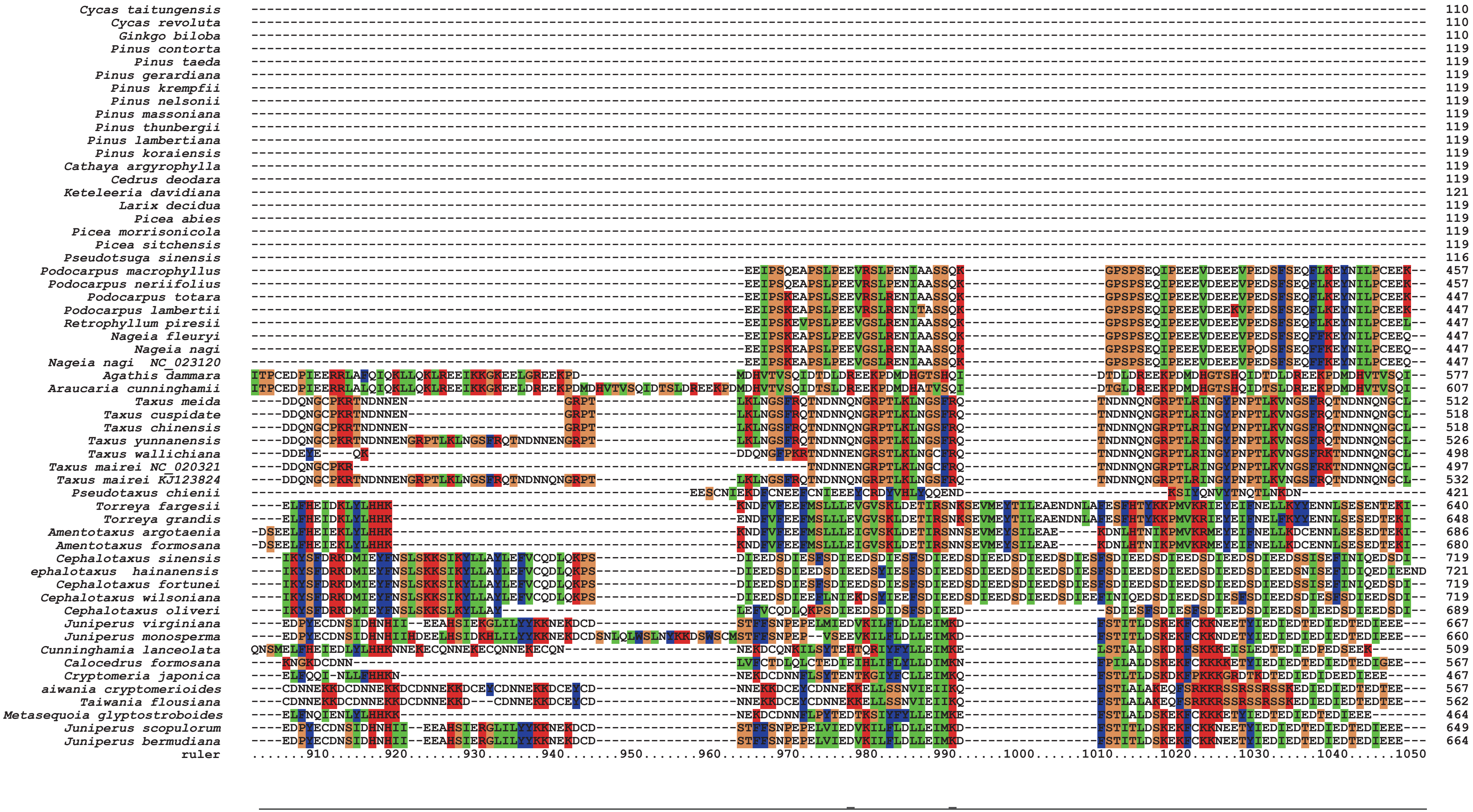

Supplementary Figure 1-8

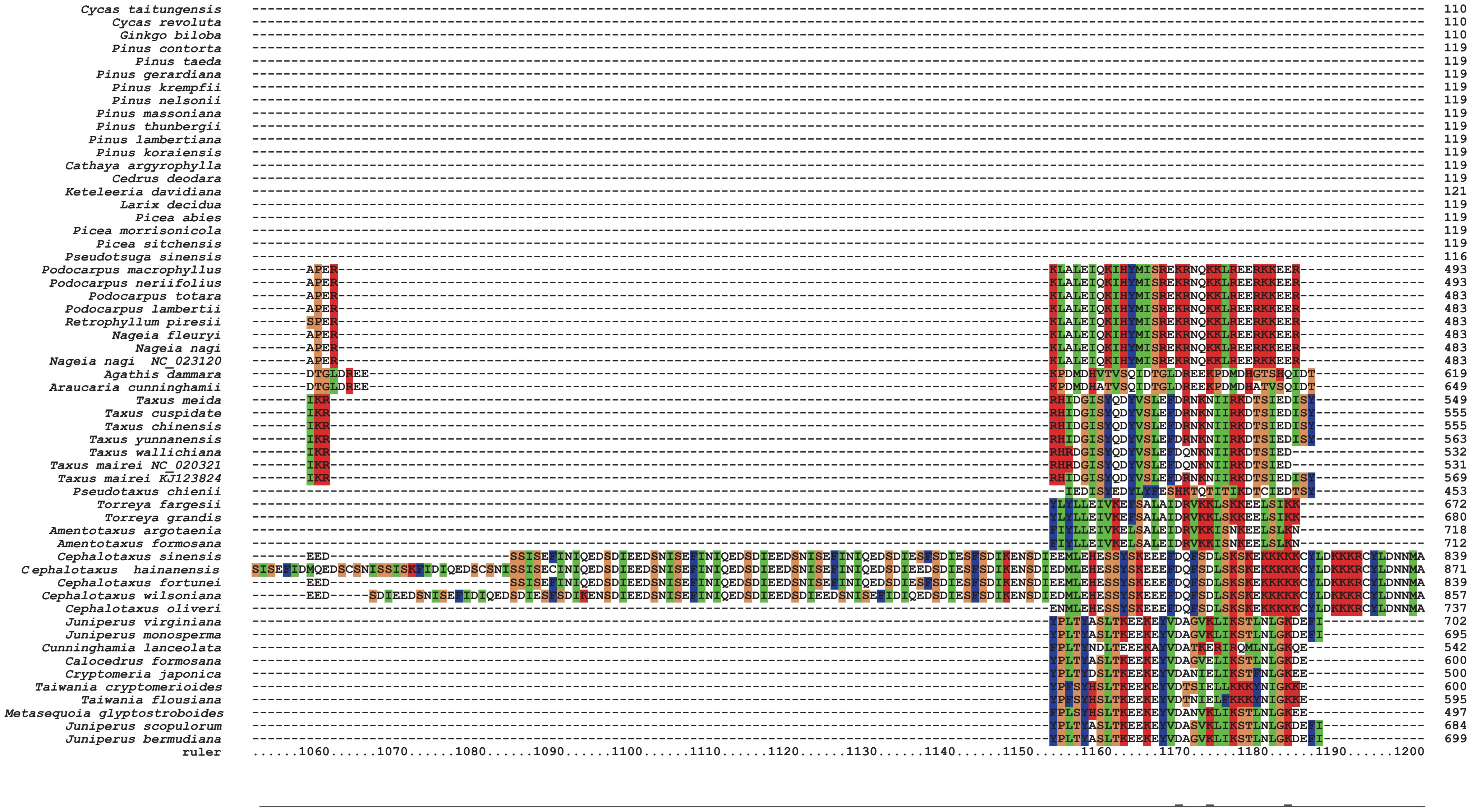

Supplementary Figure 1-9

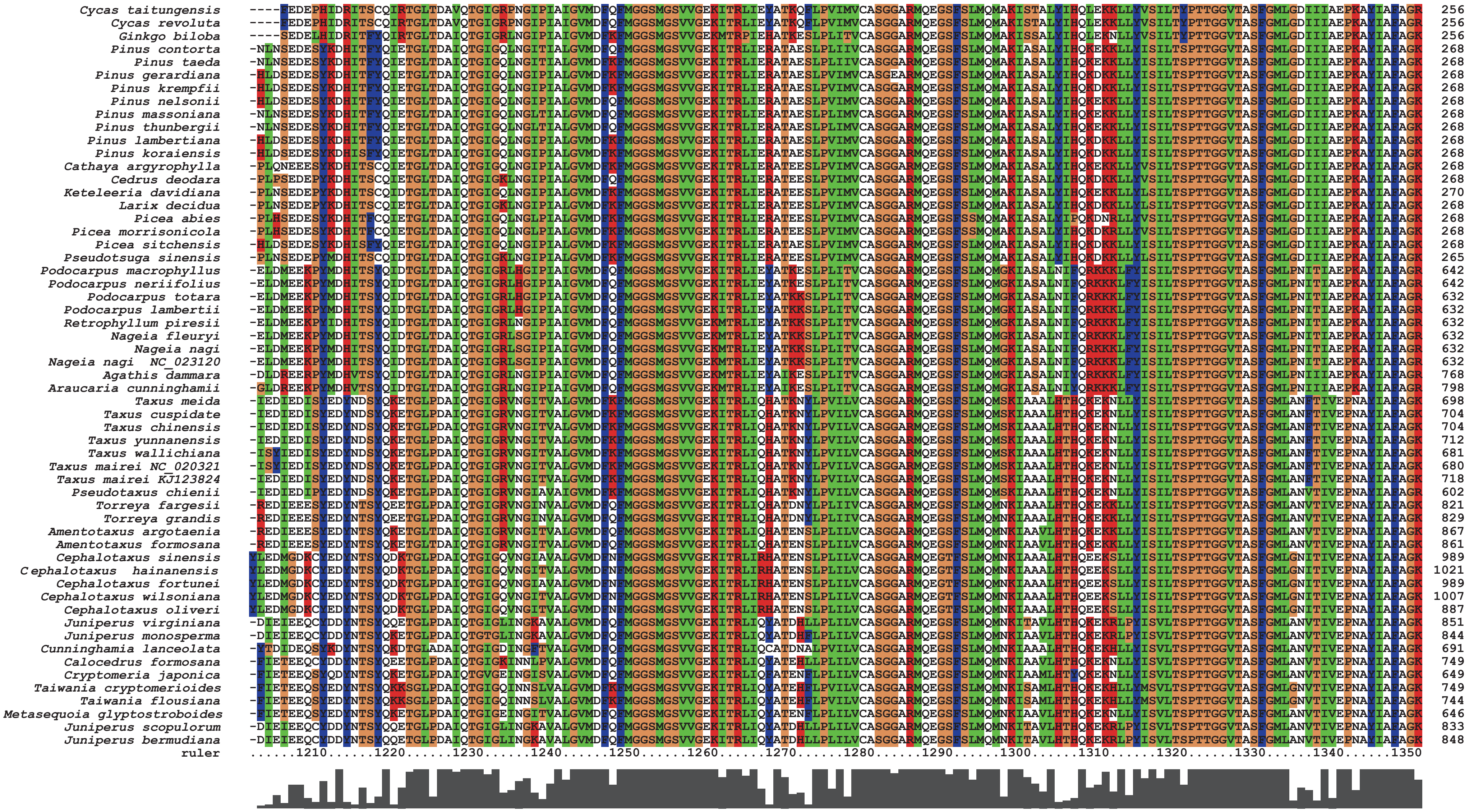

Supplementary Figure 1-10

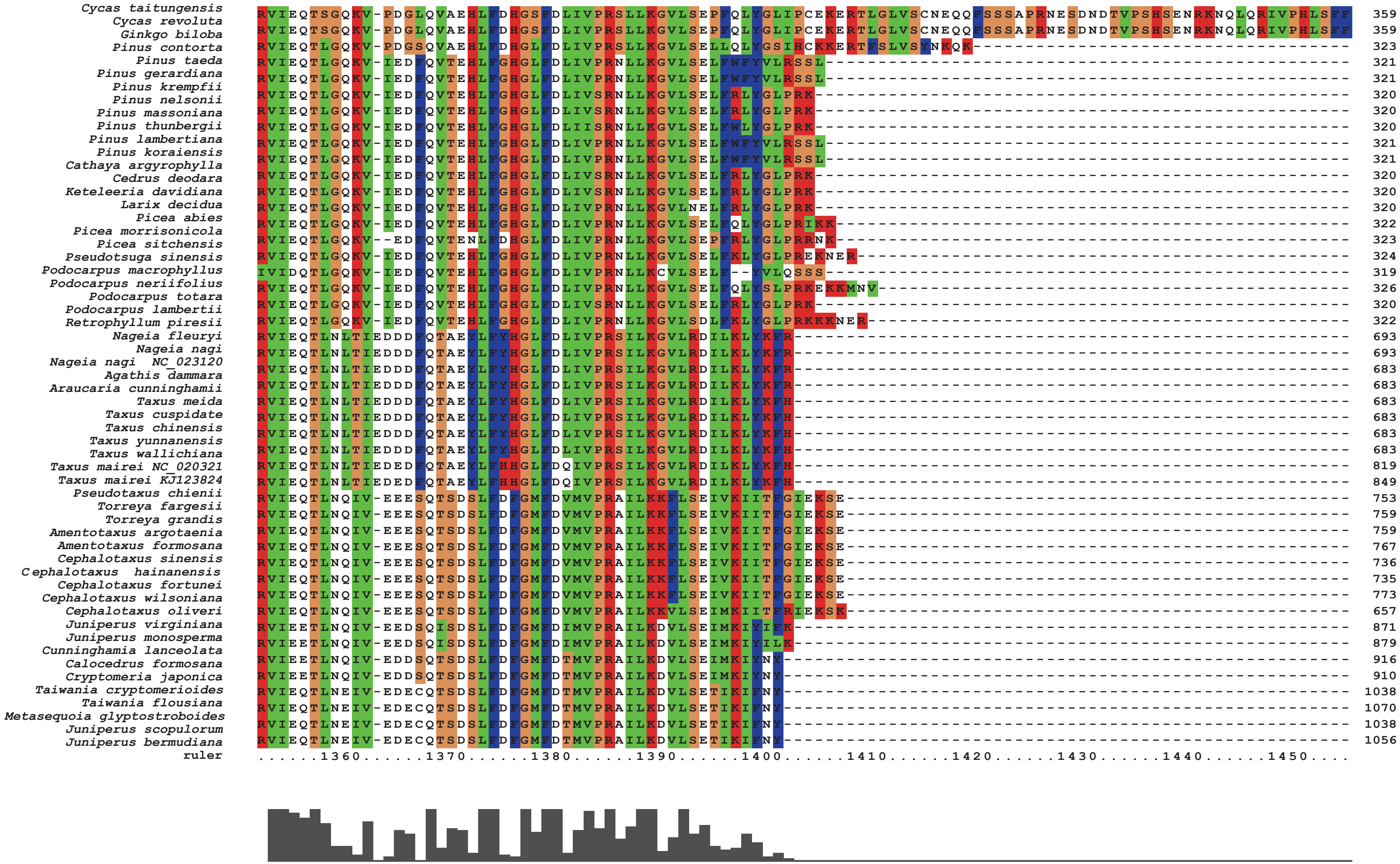

Supplement: Supplementary file 4 [file Image_1.PDF]
